# A Cluster‐Based Deep Learning Model Perceiving Series Correlation for Accurate Prediction of Phonon Spectrum

**DOI:** 10.1002/advs.202406183

**Published:** 2024-10-18

**Authors:** Chao Liang, Yilimiranmu Rouzhahong, Shunwei Yao, Junhao Liang, Chunlin Yu, Biao Wang, Huashan Li

**Affiliations:** ^1^ School of Physics Sun Yat‐Sen University Guangzhou 510275 China; ^2^ School of Materials Science and Engineering Dongguan University of Technology Dongguan 523808 China; ^3^ Guangdong Provincial Key Laboratory of Magnetoelectric Physics and Devices School of Physics Sun Yat‐sen University Guangzhou 510275 China; ^4^ Center for Neutron Science and Technology School of Physics Sun Yat‐sen University Guangzhou 510275 China

**Keywords:** cluster capsule representation, machine learning, predictive model, series correlation, spectral property

## Abstract

The spectral properties are the most prevalent continuous representation for characterizing transport phenomena and excitation responses, yet their accurate predictions remain a challenge due to the inability to perceive series correlations by existing machine learning (ML) models. Herein, a ML model named cluster‐based series graph networks (CSGN) is developed based on the dynamical theory of crystal lattices to predict phonon density of states (PDOS) spectrum for crystal materials. The multiple atomic cluster representation is constructed to capture the diverse vibration modes, while the mixture Gaussian process and dynamic time warping mechanism are compiled to project from clusters to PDOS spectrum. Accurate predictions of complicated spectra with multiple or overlapping peaks are achieved. The high performance of CSGN model can be attributed to the pertinent feature extraction and the appropriate similarity evaluation, which enable the natural perception of structure‐property relation and intrinsic series correlations as confirmed in the predictive results. The transferable and interpretable CSGN model advances ML predictions of spectral properties and reveals the potential of designing ML methods based on physical mechanisms.

## Graphical abstract

A machine learning model (CSGN) based on the cluster graph and mixture Gaussian process is developed to predict phonon density of states (PDOS) spectrum for crystal materials. Accurate predictions of complicated spectra with multiple or overlap peaks are achieved, owing to the intelligent perception of structure‐property relation and intrinsic series correlations.

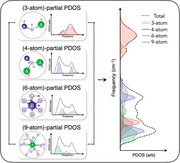

## 1 Introduction

Spectral properties serve as the most prevalent continuous representation, influenced by both intrinsic physical‐chemical features and external activating conditions.^[^ ^1^ ^]^ It can reflect the diverse condensed states, transport phenomena, and excitation responses in physical, chemical, and biomaterial systems.^[^ ^2^ ^]^ The large‐scale prediction of spectral properties based on machine‐learning (ML) model would noticeably accelerate the material design and phenomenon interpretation.^[^ ^3^ ^]^ Compared to the prediction of single‐value properties that focuses on the precise mapping to target properties, spectrum prediction is a much more challenging problem owing to the additional complexity of representing a continuous target function and capturing the intrinsic correlation between different parts of the spectrum.^[^ ^4^ ^]^

Recently, the predictions of fragmentation and molecular spectra from the mass spectrum have been accomplished by various ML algorithms.^[^ ^5^ ^]^ Considering that each fragment/molecule is related to a single peak within the input sequence, such prediction of the discrete spectrum is essentially a single‐value prediction task. The progress in predicting spectral‐type properties is currently restricted in material science, wherein only a few ML models were developed to predict the phononic and electronic density of states (DOS). The Euclidean neural network (E(3)NN) model applied graph‐based representation to predict PDOS, which straightly transferred the single‐value predictive model into the spectral property prediction.^[^ ^6^ ^]^ The Mat2Spec model improved the PDOS prediction by exploring different loss functions (MAE, KL divergence, and Wasserstein distance) and combining them with contrastive learning.^[^ ^7^ ^]^ The physically informed ML model promoted the electron DOS prediction by designing the mixed loss function based on spectral geometric properties, statistical features, and physical features.^[^ ^8^ ^]^

Despite that the upgraded loss functions have substantially improved the continuity of target functions, the overall prediction quality of spectral properties is far from satisfactory, with the prediction error sharply increasing with system complexity.^[^ ^9^ ^]^ This can primarily be ascribed to the failure to perceive the intrinsic correlation within a spectrum and evaluate the similarity between different spectra. Specifically, the intrinsic correlation between excitations at various energetic scopes within a PDOS spectrum originates from the distinct vibration modes associated with the same vibrational cluster.^[^ ^10^ ^]^ Each phonon spectrum is contributed by multi‐scale vibrational clusters and thus possesses complicated energetic correlations that can be partially transferred to other materials with similar vibrational clusters.^[^ ^11^ ^]^ Such correlations are impossible to perceive by applying general deep neural networks on the crystal graph.^[^ ^12^ ^]^ Besides, peak features are the critical information of a spectrum, yet the current loss functions based on Lp norm distances emphasize the accumulative single‐point errors rather than the deviation of peak features, leading to an inaccurate gradient for the prediction of spectra with multiple or overlap peaks.^[^ ^13^ ^]^ To overcome the above challenges, it is essential to design a spectrum‐oriented ML model based on the continuous representation and the underlying structure‐property relationship.

Herein, we developed a spectrum prediction model named cluster‐based series graph networks (CSGN) to perceive series correlations and to identify spectral patterns of PDOS. The model comprises a phonon representation module describing the contributions of multi‐scale atomic clusters, as well as a spectral predictive module based on mixture Gaussian process (MGP) and dynamic time warping (DTW) mechanism,^[^ ^13^, ^14^ ^]^ which is an advanced statistical method used primarily for measuring similarity between two temporal series with complex evolutionary features. The results verified that the series correlations have been successfully learned by the CSGN model by projecting each peak of PDOS on cluster vibrations with our continuous spectral representation method. Taking advantage of the prior physical information, the CSGN model achieves state‐of‐the‐art (SOTA) prediction performance and interpretability comparable to the atomic‐scale simulations, which provides a potential architecture for exploring complicated spectral properties in multidisciplinary science and discovering outstanding thermal conductive materials.

## 2 Results

### 2.1 Development of CSGN to Perceive Series Correlation

**Figure 1:**
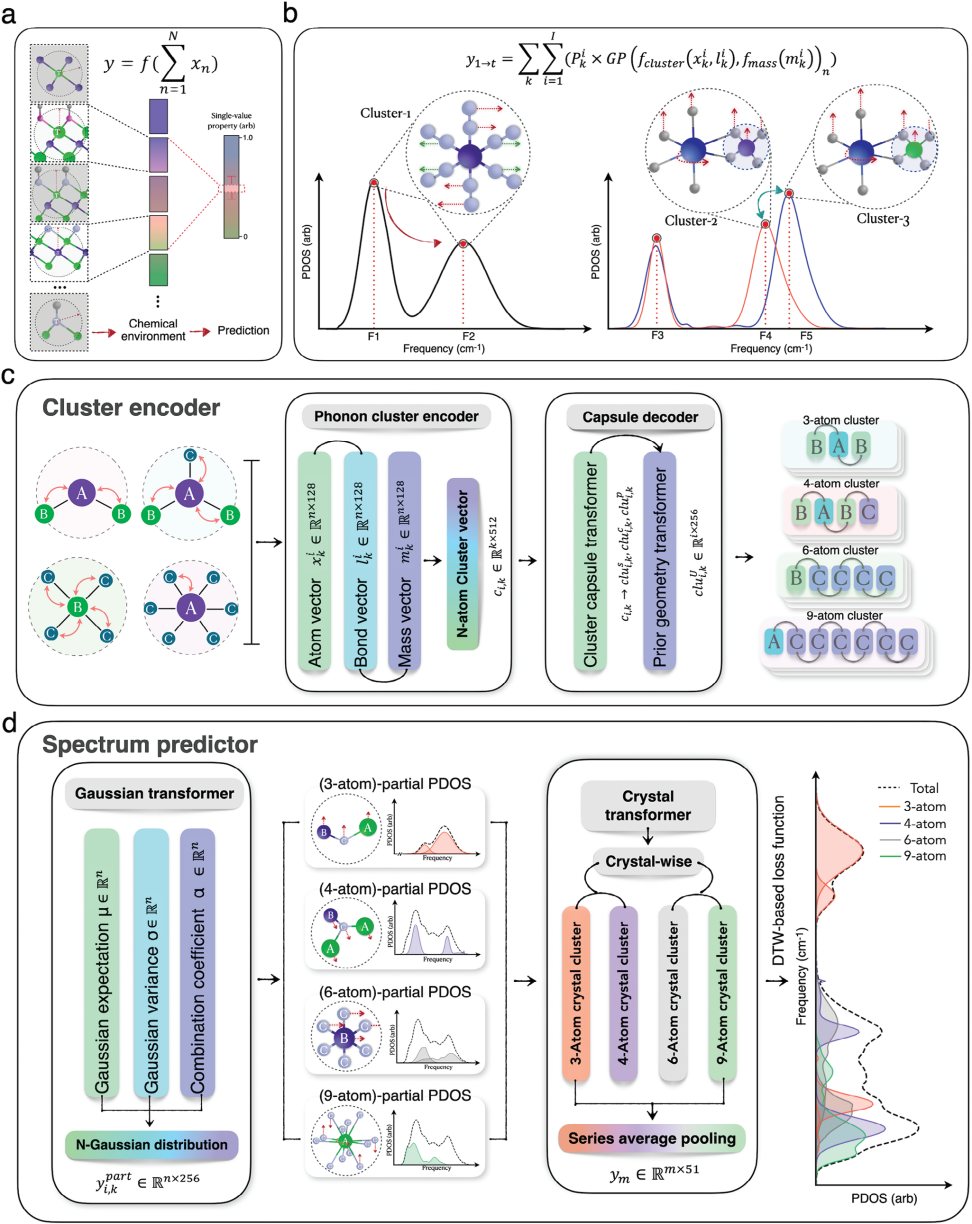
Development of the CSGN (cluster‐based series graph networks) model to percept series correlation and to predict PDOS (phonon density of states). a) Schematic illustration of single‐value property prediction. b) Schematic illustration of spectral pattern identification via multi‐atom cluster representation, where F_1_ to F_5_ are frequency values for the main peaks induced by different clusters. c) Schematic framework of the cluster encoder block to generate cluster representation relevant to multi‐scale vibrational excitation, where $x_{k}^{i}$, $l_{k}^{i}$, and $m_{k}^{i}$ are atom, bond, and mass vectors for the *i*‐center atom, *c*_*i*,*k*_ is the *i*‐center atom cluster graph with *k* scale. d) Schematic framework of the spectrum predictor block to project cluster representation on mixture Gaussian spectra.

Typical ML models in material science for predicting single‐valued property rely on the construction of nonlinear mapping $y=f(\sum_{n=1}^{N}x_{n})$ between the microscopic structure and target property *y*, wherein *x_n_* is the chemical environment of the *n*th atom in the primitive cell (**Figure** **1**a). Such models cannot be employed to appropriately predict PDOS spectrum given their inappropriate perception of series patterns and intrinsic correlations in continuous energetic space. Considering that a PDOS spectrum *y*_1 → *t*_ is composed of various vibrational excitations induced by multi‐scale atomic clusters, several peaks in different energy scopes may arise from the same cluster, while similar clusters in different materials would lead to analogous peaks in spectrum (Figure 1b).

Accordingly, we modified the prediction model for PDOS as the weighted summation of cluster contributions, via the projection of cluster graph to mixture Gaussian function.

(1) $$\begin{array}{c} F\left(y_{1\to t}|x,\;l,m,n\right)=\sum_{k}\sum_{i=1}^{I}\left(P_{k}^{i}\times GP{\left(f_{cap}\left(f_{cluster}\left(x_{k}^{i},l_{k}^{i}\right),\;f_{mass}\left(m_{k}^{i}\right)\right)\right)}_{n}\right) \end{array}$$

wherein $x_{k}^{i}$ denotes the *k*‐scale vibrational clusters with the *i*th atom serving as center atom (each cluster containing *k*‐1atoms), $\;l_{k}^{i}$ and $m_{k}^{i}$ indicates the relevant bond configuration vector, and mass vector, and $P_{k}^{i}$ is the superposition weight of clusters after n‐order Gaussian process. The nonlinear function *f_cluster_* generates a cluster graph based on structural features, *f_mass_* projects the mass information on cluster graph to reflect the dependence of vibrational frequency on atom mass, the capsule transformer *f_cap_* refines the cluster representation by perceiving symmetry information. The *GP*(·)_*n*_ of MGP^[^ ^14^ ^]^ generates a continuous spectrum in energy space by linearly superimposing *n* independent Gaussian distributions to represent the multiple vibrational modes of each cluster. The hypermeter *n* = 10 is set to exceed the maximum number of vibrational modes that can be generated by each cluster, with the unnecessary peaks automatically eliminated during training.

The spectrum prediction model was concretized by establishing the cluster encoder (CE) block (Figure 1c) and the spectrum predictor (SP) block (Figure 1d). The objective of CE block is to represent the multi‐scale vibrational clusters and to encode the physical characteristics relevant to vibrational excitation. For each cluster, the cluster graph was built based on the standard atomic chemical environments $x_{k}^{i}$ via representing the interaction between surrounding atoms and bonds within a cluster. Additional features including chemical bond configuration $l_{k}^{i}$ and atom mass distribution $m_{k}^{i}$ were then incorporated via concatenation operation. The cluster graphs for all kinds of clusters with 3, 4, 6, and 9 atoms iterating over the atoms in primitive cell were concatenated to form a material graph (the cluster sizes were designed to encompass diverse configurations with symmetry and to reach desired prediction performance as shown in Figure S1, Supporting Information). The double counting issue for vibrational modes regarding clusters with various sizes and center atoms was automatically addressed through training the $P_{k}^{i}$ parameter of each cluster, wherein the cluster weights can be appropriately assigned as validated in Section S1 (Supporting Information). The cluster representation *c*_*i*,*k*_ of the *i*th atom with *k*‐1 neighbor atoms is composed of three feature vectors $clu_{i,k}^{s},clu_{i,k}^{c}$, and $clu_{i,k}^{p}$, which are spatial transformation, chemical environment, and presence weight of each cluster capsule representation. The capsule transformer *f_cap_* was implemented to encode the geometric transformations into the cluster representation, which enables the identification of equivalent clusters stemming from crystal symmetry and the appropriate evaluation of similarity between different clusters.^[^ ^15^ ^]^ During the network training, the capsule transformer intelligently found the spatial transformations that maximize the similarity of chemical environments among different clusters and updated the chemical environments based on cluster similarity.

In order to predict diverse phonon spectra in continuous energy space, we constructed the SP block. The MGP was implemented on individual cluster graphs to generate the partial PDOS $y_{i,k}^{part}$ for the *i*‐center atom cluster with *k*‐1 atom via the Gaussian transformer module of the SP block. The partial PDOS of all clusters within a material was then merged, wherein the $y_{i,k}^{part}$ was multiplied by the related cluster weight $P_{k}^{i}$ (obtained from the $clu_{i,k}^{p}$ after training). The PDOS *y*_1 → *t*_ of the target material was eventually obtained by linear superposition of all partial PDOS through series average pooling layer. The DTW function based on statistical mechanism was employed to evaluate the distinction between predicted and ground truth spectra by quantifying the similarity of the geometric features of two sequences.^[^ ^13^ ^]^ The loss function was set as the summation of DTW discrepancy and mean absolute errors (MAE). This shape‐aware loss function based on DTW discrepancy is capable of perceiving complex shift or dilatation of multiple and overlap peaks, and thus can precisely assess the deviation of peak distribution and peak shape in the predicted spectrum compared to its ground truth counterpart.

The performance of CSGN was examined by the database composed of 1523 crystal materials, which was obtained from the Materials Project (MP) database and predicted by DFT calculations.^[^ ^16^ ^]^ The crystal database was divided into the training, validation, and testing datasets at a ratio of 8:1:1. The CSGN models were developed using the Python 3.8 and TensorFlow 2.15 framework.^[^ ^17^ ^]^ Detailed algorithm architecture, data structure, mixed Gaussian processes, loss function selection, equivariance perception of cluster encoder block, and training process are documented in Sections S1 and S2 (Supporting Information).

### 2.2 Microscopic Vibrational Origin of Phonon Spectrum Learned by CSGN

**Figure 2:**
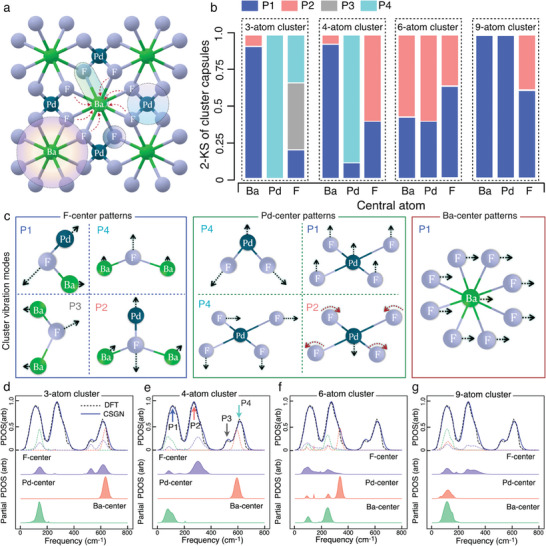
Microscopic interpretability analysis for PDOS prediction via the CSGN model. a) Schematic illustration of the multi‐scale clusters with different central atoms, wherein the cluster domains and atomic interactions are denoted by shaded regions and red arrows, respectively. b) Projected contributions of different clusters to the four PDOS peaks (P1, P2, P3, and P4 are denoted by arrows in blue, orange, gray, and green respectively in panel e) in representative BaPdF_4_ crystal from the CSGN model. The lengths of color bars display the normalized 2‐sample Kolmogorov–Smirnov (2‐KS) calculated between the partial spectra of specific clusters and the four spectral peaks (P1 to P4). c) Schematic illustration of representative phonon modes extracted from the four peaks (P1 to P4) of the BaPdF_4_ phonon structures, with the arrows indicating the vibrational directions. d–g) Decomposition of the predicted PDOS spectrum for BaPdF_4_ crystal on individual clusters with various atom numbers (3, 4, 6, and 9) and central atom types. The blue solid and black dashed lines present the predicted and ground truth spectra, respectively. The purple, red, and green shaded regions denote the contributions from clusters with F, Pd, and Ba centers.

Since the ML framework was developed based on the dynamical theory of crystal lattices, it would be expected that our CSGN model not only offers accurate prediction of PDOS spectrum, but also appropriately identifies the origin of each peak to vibrational clusters.^[^ ^18^ ^]^ To verify such a premise, we compared our predicted PDOS spectra and the extracted cluster contributions to the ground truth data obtained by DFT simulations in previous studies (PDOS from the MP database, and vibration modes from the Phonon website). The comprehensive analyses of various materials demonstrated the efficacy of CSGN model, and the detailed results for the BaPdF_4_ crystal will be discussed in this section (**Figure** **2**). The BaPdF_4_ crystal was selected as a representative material owing to its relatively complex spectrum, diverse vibrational clusters, and high crystal symmetry that facilitates the interpretation of structural‐property mapping (Figure 2a).

The PDOS spectrum of BaPdF_4_ crystal contains four main peaks (as denoted by arrows in Figure 2e), which represent the varying activation of vibration modes under different energy excitations. The predicted spectrum precisely matches the ground truth spectrum as shown in Figure 2d. To investigate the contributions of vibrational clusters on each peak, we extracted the predicted partial PDOS spectra related to the *k*‐atom (*k *= 3, 4, 6, and 9) clusters with different types of atoms serving as cluster centers (there is only one inequivalent atom in each element type). The normalized 2‐sample Kolmogorov–Smirnov (2‐KS) was then calculated between the partial PDOS spectra and the four spectral peaks (Figure 2b). The null hypothesis is that the two distributions are identical, and the alternative hypothesis is that they are not identical, which is justified by the low p‐value. The detailed calculated 2‐KS results are documented in Table S1 (Supporting Information). The results suggest that the P4 pattern with high excitation energy arises from the contributions of 3, 4‐atom Pd‐center clusters and 3‐atom F‐center clusters (Figure 2d,e), consistent with the truth phonon modes in the same energy scope obtained from the Phonon website (Figure 2c). In addition, the 9‐atom vibrational clusters contribute only to the P1 and P2 patterns (Figure 2g), which are in accordance with the true energetic distribution of 9‐atom phonon modes (Figure 2c) and can be explained by the excitation of cluster with a large mass in low energy range. In all, the decomposition of PDOS predicted by the CSGN model (Figure 2d–g) aligns well with the multi‐scale cluster vibrational modes (Figure 2c).

The above spectrum decomposition also justifies the underlying mechanism of the CSGN model to perceive intrinsic correlations within a spectrum. In particular, the 3‐atom F‐center cluster possesses just one configuration in BaPdF_4_ crystal, yet contributes to three PDOS peaks with distinct excitation energies (Figure 2d). This can be ascribed to the existence of three different vibration modes (P1, P3, and P4) regarding the same cluster as revealed by the ground truth data (Figure 2c). The projections of a single cluster on multiple peaks are also observed and examined for the Pd‐center and Ba‐center clusters (Figure 2f), which indicate that the complex energetic correlations have been perceived by the CSGN model. Such intrinsic correlation among different peaks learned from the training set can be naturally transferred to the predicted spectra of new materials containing similar vibrational clusters.

### 2.3 Global Trends of PDOS Spectrum Distribution Reflected by CSGN

**Figure 3:**
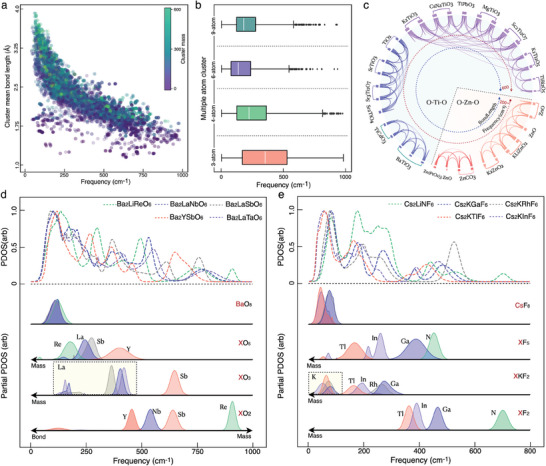
Global analysis of the physical trends in PDOS (phonon density of states) spectrum distribution predicted by the CSGN (cluster‐based series graph networks) model. a) Correlation analysis among cluster mean bond length, cluster mass, and major peak frequency of all clusters in the phonon datasets, with the cluster mass indicated by colors of nodes. b) Dependence of major peak frequency on cluster scale via the box plot. The 3‐, 4‐, 6‐, and 9‐atom cluster groups are presented in dark green, purple, light green, and orange, respectively. The box plots show maximum and minimum values (whiskers), upper and lower quartiles (box boundaries), and median values (horizontal lines). c) Cluster‐based correlation analysis of partial PDOS for 36 materials via the chord plot. Each node in the outermost ring represents a material containing either O–Ti–O or O–Zn–O clusters. The color and thickness of chords denote the similarity of partial PDOS generated by the same clusters in different materials. The inner circles in red and blue dashed lines illustrate the variation trends of vibrational frequency and bond length, respectively. d,e) Comparative analysis of phonon spectra in two representative material groups, including Ba‐X‐Y‐O (Ba_2_LiReO_6_, Ba_2_LaNbO_6_, Ba_2_LaSbO_6_, Ba_2_YSbO_6_, and Ba_2_LaTaO_6_) and Cs‐X‐Y‐F (Cs_2_LiNF_6_, Cs_2_KGaF_6_, Cs_2_KRhF_6_, Cs_2_KTlF_6_, and Cs_2_KInF_6_). The PDOS of each material is presented at the top with dashed line, and the partial PDOS of relevant clusters is shown at the bottom. The colors of dashed lines and shaded regions are consistent and denote the corresponding material. The arrows indicate the increasing directions of cluster mass or bond length.

The appropriate perceptions of the structural similarity and the consequential spectral correlation among diverse crystal materials are essential to the accurate prediction of PDOS spectrum. We comprehensively analyzed the global trends of the predicted PDOS spectra and the relevant material features to justify the efficacy of our CSGN model based on cluster representation. For individual clusters in all materials, the frequencies of the main peak in the predicted partial PDOS spectra were extracted and collectively presented in **Figure** **3**a. The results imply that our cluster representation is capable of generating diverse PDOS spectra to continuously cover the entire energy range of relevant phonon modes, with the entire data documented in Section S4 (Supporting Information). The overall trend that the peak frequency increases with decreasing cluster mass and average bond length can be found in the peak distribution, which is consistent with the vibrational origin of phonon spectra. To quantify the strain effect in our model, we have conducted two series of additional calculations for predicting PDOS with material datasets in different strain conditions as shown in Figures S5 and S6 (Supporting Information). The test results verified the robustness of CSGN model to strain effect and indicated that the CSGN model successfully learned the underlying structure‐property relation. From the projection of peak distribution, the clusters with different sizes contribute to distinct energy ranges, with the average frequency declining with cluster scales (Figure 3b).

To unveil the impact of crucial material features (including element type, bond configuration, and condensed phase) on evaluating cluster similarity and predicting partial PDOS, we sampled the O–Ti–O and O–Zn–O clusters in 36 representative materials to calculate the correlation of partial PDOS via the chord diagram (Figure 3c). These two 3‐atom clusters were chosen for their symmetric structure and rich embedding crystal materials. In the chord diagram, the nodes at the edge represent different clusters from distinct materials, and the connecting lines indicate the similarity between the partial PDOS of different cluster nodes. The Pearson coefficients are presented by the line thickness, and each group of clusters with coefficients over 0.8 is highlighted by the same color. The O–Ti–O and O–Zn–O clusters generate three and two groups of similar partial PDOS in the relatively high and low energy ranges respectively, wherein each group contains various types of crystal materials. The materials with analogous chemical components present in the same group, and those with identical stoichiometry but different phases possess the largest correlations, which confirm that the similarity of clusters among materials has been appropriately learned by the CSGN model. In addition, the clusters within the same group exhibit analogous bond lengths, while noticeable differences in bond lengths are observed between different groups, suggesting that the impact of varying bond lengths on partial PDOS sufficiently reflects those of the global chemical environment. The diverse partial PDOS associated with the simple O–Ti/Zn–O clusters can thus be attributed to the appropriate perception of delicate change in cluster microscopic structure and the subsequent mapping on Gaussian distributions.

The underlying structure‐property relationship learned by the CSGN model was further probed by scrutinizing the partial PDOS of related clusters within two representative material groups (BaXYO and CsXYO). As validated in Figure S8 (Supporting Information), the predicted PDOS of all these materials accurately matches the ground truth spectra. The top panels of Figure 3d,e present the predicted total PDOS for each material, and the bottom panels illustrate the partial PDOS of the embedding clusters with similar chemical environments. Consistent with the harmonic vibration model in condensed matter physics, our results suggest that the vibrational frequency critically depends on the mass of motional atoms, bond lengths, and restoring force constant.

For clusters with identical structures but in different crystals (groups BaO_8_, CsF_8,_ and LaO_3_), the partial PDOS are similar, and the peak frequency slightly drops with increasing bond lengths. Given that restoring force constant is positively correlated to the second derivative of energy with respect to interatomic distance^38^, the increasing bond length would diminish the restoring force constant and thus lead to lower vibrational frequency. Regarding the clusters containing different center atoms but with the same surrounding atoms, the partial PDOS spread in a much broader frequency range. In most cases (group XO_5_, XO_3_, XF_5_, XKF_2_, and XF_2_), the peak frequency substantially decreases with increasing cluster mass (atom mass: Re> La> Sb> Y, Tl> In> Rh> Ga> N), as expected from the inverse proportion relation between mass and vibrational frequency. However, for the cluster group with relatively small variation in cluster mass, the impact of restoring force constant becomes more important, and the opposite trend with cluster mass could be observed. Within the group XO_2_, the PDOS peaks of the ReO_2_ and SbO_2_ clusters with ionic bonds are distributed in the relatively higher frequency range compared to the NbO_2_ and YO_2_ clusters with covalence bonds. The frequency differences within each bond type (NbO_2_> YO_2_, SbO_2_> ReO_2_) can be further explained by the varying restoring force constants as reflected in their bond energies (Sb‐O:200–300 kJ mol^−1^. Re‐O:300–400 kJ mol^−1^, Nb‐O:600–800 kJ mol^−1^, Y‐O: 300–400 kJ mol^−1^). For the cluster groups possessing multiple PDOS peaks, distinct trends can be observed for different peaks. While the second PDOS peak within the group XKF_2_ noticeably shifts with cluster mass, the frequency of the first peak remains almost unchanged, probably because the center atom is static in relevant vibration mode. Detailed calculated results, physical, and chemical information of the selected materials are documented in Section S5 (Supporting Information).

### 2.4 Performance Analysis of CSGN

The performance of our CSGN model in predicting PDOS was examined through the comparison with SOTA models including Mat2Spec and E3NN.^[^ ^6^, ^7^ ^]^ The CSGN model possesses a MAE of 0.061 for the normalized testing set, which is 21.8% and 41.9% lower than those of the Mat2Spec (0.078) and E3NN (0.105) models by testing the same datasets with lower computing cost. More importantly, the prediction of complicated PDOS spectra with multiple and overlap peaks remains a challenge for existing algorithms, but can be generally achieved by the CSGN model.

**Figure 4:**
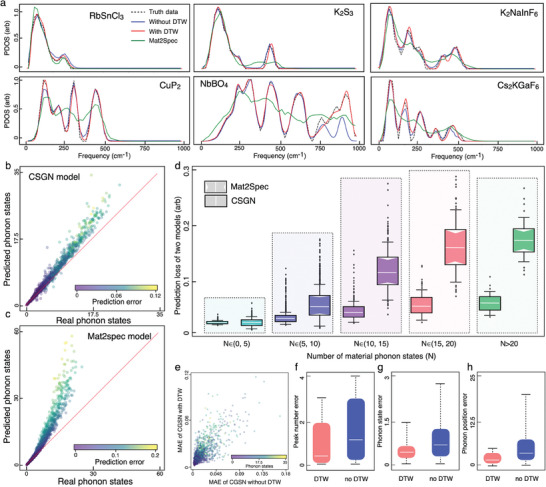
Prediction performance of PDOS (phonon density of states) spectrum via the CSGN (cluster‐based series graph networks) model. a) Comparison of predictive PDOS spectra with different models for six representative materials. The spectra predicted by the CSGN model without DTW (dynamic time warping) kernel, the CSGN model with DTW, and the Mat2Spec model are shown in the blue, red, and green solid lines respectively, while the ground truth spectra are presented in the black dashed lines. b,c) Prediction performances of phonon state numbers by the CSGN and Mat2Spec models, with the mass of primitive cell illustrated by the color of node. d) Comparison of prediction loss distributions between the CSGN and Mat2Spec models for material systems with different phonon state numbers. The box plots show maximum and minimum values (whiskers), upper and lower quartiles (box boundaries), and median values (horizontal lines). e) Comparison of predictive error distributions between the CSGN models with and without DTW kernel, with the phonon state number illustrated by the color of node. f–h)The effectiveness analysis of DTW kernel for detecting prior physical features of phonon spectrum, including peak number, phonon states, and phonon position.

Six representative materials (RbSnCl_3_, K_2_S_3_, K_2_NaInF_6_, CuP_2_, NbBO_4_, and Cs_2_KGaF_6_) within the testing set were chosen to illustrate the prediction performance (**Figure** **4**a), wherein the first five materials are consistent with the selections in the publications of the Mat2Spec and E3NN models. For relatively simple PDOS spectra shown in the first row, both of the Mat2Spec (green line) and CSGN (red line) models appropriately predicted the frequencies and shapes of the main peaks. The satellite peaks overlapping with the main peaks in K_2_S_3_ and K_2_NaInF_6_ can be adequately distinguished by the CSGN model yet not by the Mat2Spec model. For complicated PDOS spectra with multiple peaks spreading in broad energy range as shown in the second row (CuP_2_, NbBO_4_, and Cs_2_KGaF_6_), the Mat2Spec model captured the overall distributions, yet failed to predict the peak numbers and peak positions. The error of the Mat2Spec model is especially large in the high‐frequency range as illustrated in the spectrum of NbBO_4_, probably due to the insufficient size of dataset. In contrast, the CSGN model can achieve transferable prediction to generate high‐quality PDOS spectra with accurate peak distribution and peak strength in the full energy range, as validated by testing materials without similar components in the training set. More comparison results can be found in Figure S9 (Supporting Information).

The total number of phonon states defined as the integral of PDOS spectrum is an intrinsic material feature, which predictive error is technically related to the accumulative MAE of PDOS spectrum. Materials with larger phonon state numbers are likely to possess more complicated crystal structure, larger diversity of PDOS pattern, and broader range of vibration frequency. The global performances of the CSGN and Mat2Spec models in all materials were measured by comparing the predicted numbers of phonon states to the ground truth values. It turns out that the predictive error of Mat2Spec model gradually increases with the number of phonon states (Figure 4b), while the accurate prediction of phonon state number by the CSGN model is maintained in the entire material space (Figure 4c). Quantitative comparison of model performance was conducted on material groups with different phonon state numbers. From the box plots of MAE distributions (Figure 4d), the prediction performance of CSGN model exceeds the Mat2Spec model in material systems regardless of spectrum complexity, and the improvement become significant for systems with relatively large phonon state number (N >10).

The desired performance of CSGN model can be attribute to the improvement of spectrum representation (including cluster graph and Gaussian generator) and loss function. The impacts of these factors were analyzed by comparing the predictive errors of the CSGN model with and without the DTW loss function. The MAE of PDOS prediction by the CSGN model without the DTW loss function is mainly distributed in the range of (0, 0.066), which is lower than the (0, 0.078) of Mat2Spec model and higher than the (0, 0.061) of CSGN model with the DTW loss function (Figure 4e). The predictive errors of peak number (Figure 4f), phonon states (Figure 4g), and peak frequency (Figure 4h) were reduced by 45.2%, 58.7%, and 62.1% via implementing the DTW loss function. As illustrated in Figure 4a, despite that the CSGN model is capable of accurately predicting the peak positions in most materials, the DTW loss function plays an important role in refining the peak shapes especially for complicated spectra. The above results suggest that the collaboration of advanced spectrum representation and loss function is crucial for the CSGN model.

## 3 Discussion

The above comprehensive analyses demonstrated the efficacy of our CSGN model and enabled us to interpret its underlying mechanism. The key idea is to develop a transparent ML model based on the dynamical theory of crystal lattices for accurately encoding the structural/spectral diversity and similarity,^[^ ^18^ ^]^ as well as naturally perceiving the structure‐property relation and intrinsic spectral correlation, which is accomplished as follows: 1) We composed a mass decorated multiple atomic cluster representation with 40,496 cluster types to describe the rich vibration modes originating from clusters with different sizes and shapes. Such representation provides a more flexible and precise feature extraction of crystal materials compared to the traditional crystal graph comprised of spherical atomic environments with a uniform fixed radius. 2) We designed a spectral prediction method including the mapping of cluster graphs on mixture Gaussian functions and the linear superposition of predicted partial PDOS toward the predictive spectrum. This is consistent with the physical scenario that the PDOS is a superposition of all phonon contributions, and the peak frequencies are determined by the relevant vibration modes. As verified by the prediction results, the CSGN model appropriately learned the mapping from clusters to PDOS peaks and the frequency shift with changing chemical environment. The spectral correlations within a spectrum arising from the different vibration modes of the same cluster and the correlation across the materials arising from the similarity between vibrational clusters are thus adequately embedded in our predictive spectrum. The decomposition of PDOS spectrum into the contributions of atomic clusters also effectively increases the data size and address the insufficient database of PDOS spectrum. 3) We compiled a DTW‐based loss function to perceive the shift or dilatation of peaks and thus to appropriately evaluate the spectral differences. This overcomes the drawback of traditional Euclidean‐based function (MAE, MSE, etc.) and simple statistical (KL, WD divergence, etc.) loss functions that underestimate the prediction error due to the error cancellation for any spectrum variation with preserved integral value.

In summary, we have developed the CSGN model consisted of the cluster encoder and spectrum predictor blocks to accurately predict PDOS spectrum of crystal materials. The predictive results demonstrate that the CSGN model has successfully conducted the mapping from vibrational clusters to PDOS patterns, as well as the transformation of vibrational frequency in response to the chemical environment modification. The rational structure‐property relation learned by the CSGN model guarantees the appropriate reflection of series correlations in the predictive spectra, and thus enables the accurate prediction of complicated spectra with multiple or overlap peaks. The high performance and interpretability of the CSGN model can be attributed to both the establishment of cluster‐spectrum representation based on crystal lattices dynamical theory and the subsequent identification of pattern similarity with DTW loss function. This work not only provides a transferable ML model to improve the prediction of spectral properties but also reveals the opportunity to design advanced ML framework based on the fundamental physical mechanisms.

## 4 Experimental Section

### Framework of the CSGN

In this work, accurate predictions of materials’ spectral properties were achieved, benefited from the identification of spectrum patterns and series correlations via the CSGN model. This was accomplished through the unified training of two modules including the cluster encoder (CE) block and the spectrum predictor (SP) block.

The CE block perceived the input data of atoms and bonds to construct the multi‐scales cluster representation of crystal materials (**Table** **1**), through the hierarchical aggregation of chemical environments and the encoding of symmetric transformations. The CE block was composed of four modules including the atomic cluster module, bond distribution module, cluster mass module, and capsule‐based symmetry module. The first three modules received and extracted prior features to encode the multi‐scale cluster environments, and the last module transmits the full symmetry transformations of space groups to update the cluster representation.

**Table 1:** Algorithm 1 Cluster encoder block.

**Input**: $x_{k}^{atom}=\left(x_{k}^{1},\text{…},x_{k}^{i}\right)\in R^{i\times k\times 128}$, $x_{k}^{bond}=\left(l_{k}^{1},\text{…},l_{k}^{i}\right)\in R^{i\times j\times 128}$, $\;x_{k}^{mass}=\left(m_{k}^{1},\text{…},m_{k}^{i}\right)\in R^{i\times k}$ **Prior modules**: $f_{cluster}=\left(f_{a}\left(\cdot \right),f_{b}^{atom}\left(\right),f_{b}^{cluster}\left(\right)\right)$, *f_mass_* (·), *f_cap_* (·), **Output**: $clu_{i,k}^{s},clu_{i,k}^{c},clu_{i,k}^{p}\in R^{i\times 128},R^{i\times 128},R^{i\times 1}$
**For** *k* iterations **do**: **For** *i* iterations **do**: $c_{i,k}^{\mathrm{atom}}\leftarrow f_{a}\left(x_{k}^{i}\right)$ atom composition for each cluster $b_{i,k}^{\leftarrow }f_{b}^{atom}\left(l_{k}^{i}\right)$ bond configuration for each cluster $c_{i,k}^{\mathrm{mass}}\leftarrow f_{mass}\left(m_{k}^{i}\right)$ mass distribution for each cluster $c_{k}^{A}\leftarrow Softplus\left(c_{i,k}^{atom}\right)$ $c_{k}^{B}\leftarrow f_{b}^{cluster}\left(b_{i,k}\right)$ $c_{k}^{M}\leftarrow Softplus\left(c_{i,k}^{mass}\right)$ $c_{k}^{\mathrm{total}}\leftarrow c_{k}^{A}⊕c_{k}^{B}⊕c_{k}^{M}$ $\;c_{i,k}={\mathrm{clu}}_{i,k}^{s},{\mathrm{clu}}_{i,k}^{c},{\mathrm{clu}}_{i,k}^{p}\leftarrow f_{cap}\left(c_{k}^{\mathrm{total}}\right)$ **End for** **End for** **Return** ${\mathrm{clu}}_{i,k}^{s}={\left(clu_{1}^{s},\text{…},clu_{i}^{s}\right)}_{k}$ ${\;\mathrm{clu}}_{i,k}^{c}={\left(clu_{1}^{c},\text{…},clu_{i}^{c}\right)}_{k}$ ${\;\mathrm{clu}}_{i,k}^{p}={\left(clu_{1}^{p},\text{…},clu_{i}^{p}\right)}_{k}$

The crystal information files (CIFs) of the MP datasets are filtered by leveraging the Pymatgen^35^ interface to construct the input dataset, with the detailed information of original dataset documented in Section S1 (Supporting Information). The input dataset includes the atom composition $x_{k}^{atom}=\left(x_{k}^{1},\text{…},x_{k}^{i}\right)$, bond connection $x_{k}^{bond}=\left(l_{k}^{1},\text{…},l_{k}^{i}\right)$, and mass distribution $x_{k}^{mass}=\left(m_{k}^{1},\text{…},m_{k}^{i}\right)$ of all clusters with different center atoms *i* ∈ *N* and different scales (*k* = 3, 4, 6, and 9). For each cluster, the atom data $x_{k}^{i}$ reflects the atom number and types through the atomic embedding vectors with (*k* × 128) dimensions, the bond data reflects the bond number, connected atom types, and bond lengths through the bond embedding vectors (with *j* × 128 dimensions expanded by the Gaussian basis, *j* was the bond number of cluster), and the mass data reflects the atomic mass distribution by a vector with (*k* × 1) dimensions.

The chemical environment of each cluster was built parallelly. The atom module *f_a_* (·) generates the matrix $c_{i,k}^{atom}$ with (*k* × 192) dimensions from $x_{k}^{i}$ through the multi‐layer perceptron (MLP), which encodes the atomic interactions within each cluster and modulates the similarity between different clusters. The mass module *f_mass_* (·) builds the matrix for each cluster to highlight the dependence of vibration modes on atom mass via the MLP‐based model, with the output $c_{i,k}^{mass}$ of (*k* × 128) dimensions. The atom and mass matrices were then modulated with the *Softplus* activation function for enhancing the smoothness of material representation. The bond module consists of atom‐level $f_{b}^{atom}\left(\cdot \right)$ and cluster‐level $f_{b}^{cluster}\left(\cdot \right)$ sub‐modules, which were both MLP‐based models with outputs $b_{i,k}$ of (*j* × 192) dimensions and $c_{k}^{B}$ of (*k* × 192) dimensions. The former encodes the environment vector for each bond via receiving bond index and connected atom information, and the latter projects the information of all bonds on each atom to generate the bond matrix. The cluster chemical environment $c_{i,k}^{total}$ was obtained by concatenating the atom, bond, and mass matrices.

(2) $$\begin{array}{c} c_{k}^{total}=Softplus\left(f_{a}\left(x_{k}^{i}\right)\right)⊕\left(f_{b}^{cluster}\left(f_{b}^{atom}\left(l_{k}^{i}\right)\right)\right)⊕\left(Softplus\left(f_{mass}\left(m_{k}^{i}\right)\right)\right) \end{array}$$

The cluster chemical environment was then transformed into the cluster capsule representation $c_{i,k}=\left\{clu_{i,k}^{s},clu_{i,k}^{c},clu_{i,k}^{p}\right\}$ through the capsule encoder *f_cap_* (·) following the standard procedure developed in the SEN model.^[^ ^15^ ^]^ Such transformation refines the evaluation of cluster similarity by recognizing the preservation of cluster properties under rigid geometric transformations including rotation and reflection. The geometric transformation operator $clu_{i,k}^{s}$ and the convoluted cluster chemical environment $clu_{i,k}^{c}$ are both vectors with 128 dimensions. The presence value $clu_{i,k}^{p}$ was a variable to measure the weight of corresponding cluster.

The SP block was designed to predict diverse phonon spectrums for different material systems by receiving and decoding cluster capsule representation, as illustrated in **Table** **2**. The cluster partial PDOS $y_{i,k}$ of *i*th atom with *k*‐scale was predicted via the MGP model, which represents the activated phonon state patterns of the target cluster under continuous energy space. *f_u_* (·) was a symmetry module that propagates the geometric transformations into cluster capsules by receiving symmetry operators $clu_{i,k}^{s}$, chemical environment $clu_{i,k}^{c}$, mainly including scaling, translation, rotation, inversion reflection, and mirroring reflection transformations. With the *Softplus* activation function, capsule weights of clusters $clu_{i,k}^{p}$.were obtained Θ_*g*_() was a parametric function to calculate parameters $N(\mu_{i,k},\sigma_{i,k},\alpha_{i,k})$ of MGP via receiving updated cluster capsules, wherein µ_*i*,*k*_, σ_*i*,*k*_, α_*i*,*k*_ were respectively the internal threshold, the width of the Gaussian process, and weight parameters, and N(·) was the MLP mathematical form. The PDOS $y_{i,k}^{u}$ of each atom‐center cluster successfully obtained by sampling from the corresponding MGP function with 256 dimensions. *f_mol_* (·) was an atom‐to‐molecular transformer model that outputs crystal‐level PDOS $y_{k}^{u}$ of material under a fixed scale according to space structures with (N × 51) dimensions. *f*_ϕ_(·) was an MLP‐based model to calculate the contribution probability of crystal‐level PDOS (Φ_*k*_ ∈ (0, 1)). The total PDOS *y_m_* of material can be eventually obtained by linear superposition of all partial PDOS through series average pooling.

**Table 2:** Algorithm 2 Spectrum predictor block.

**Input**: cluster capsule representation $clu_{i,k}^{s},clu_{i,k}^{c},clu_{i,k}^{p}\in R^{1\times 128},R^{1\times 128},R^{1\times 1}$ **Mixture Gaussian transformer**: *f_u_* (·), Θ_*g*_(·), N(·), *f_mol_* (·), *f*_ϕ_(·) **Output**: $y_{m}=\left(y_{1},\text{…},y_{m}\right)\in R^{m\times 51}$
**For** *k* iterations **do**: **For** *i* iterations **do**: ${\mathrm{clu}}_{i,k}^{u}\leftarrow f_{u}\left(clu_{i,k}^{s},clu_{i,k}^{c}\right)$ ${\mathrm{clu}}_{i,k}^{p}\leftarrow Softplus{\left(clu_{1}^{p},\text{…},clu_{i}^{p}\right)}_{k}$ $\mu_{i,k},\sigma_{i,k},\alpha_{i,k}\leftarrow \Theta_{g}\left(clu_{i,k}^{u},n\right)$ $y_{i,k}\leftarrow N\left(\mu_{i,k},\sigma_{i,k},\alpha_{i,k}\right)$ $y_{i,k}^{u}\leftarrow clu_{i,k}^{p}⊗y_{i,k}$ **End for** $y_{k}^{u}\leftarrow \sum_{I}\left(y_{i,k}^{u}\right)$ **End for** $\Phi_{k}\leftarrow f_{\phi }\left(y_{k}^{u}\right)$ $y_{m}\leftarrow \sum_{k}\left(y_{k}^{u}⊗\Phi_{k}\right)$ **Return** $y_{m}=\left(y_{1},\text{…},y_{m}\right)$

## Conflict of Interest

The authors declare no conflict of interest.

## Supporting information



Supporting Information

## Data Availability

The data that support the findings of this study are available on request from the corresponding author. The data are not publicly available due to privacy or ethical restrictions.
