# Supplementary material for: A Cluster‐Based Deep Learning Model Perceiving Series Correlation for Accurate Prediction of Phonon Spectrum
Source: Adv Sci (Weinh). 2024 Oct 18;11(46):2406183. doi: 10.1002/advs.202406183 (PMC11633492; doi:10.1002/advs.202406183)
Supplement: Supplementary file 1 — Supporting Information [file ADVS-11-2406183-s001.pdf]

ADVANCED SCIENCE
Open Access

# Supporting Information

for *Adv. Sci.*, DOI 10.1002/advs.202406183

A Cluster-Based Deep Learning Model Perceiving Series Correlation for Accurate Prediction of Phonon Spectrum

*Chao Liang, Yilimiranmu Rouzhahong, Shunwei Yao, Junhao Liang, Chunlin Yu, Biao Wang* and Huashan Li**

Supporting Information

**A Cluster-based Deep Learning Model Perceiving Series Correlation for Accurate Prediction of Phonon Spectrum**

*Chao Liang, Yilimiranmu Rouzhahong, Shunwei Yao, Junhao Liang, Chunlin Yu, Biao Wang*, and Huashan Li**

## Section 1: Information of the PDOS dataset and verification of cluster representation

Consistent with the data source in previous machine learning studies on crystal materials, the dataset for predicting phonon density of states (PDOS) spectrum in this work was obtained from the Materials Project (MP) database. The dataset contains 1523 crystal materials covering all crystal systems. These materials are composed of 64 elements, which cover the entire periodic table except for the noble gases group, lanthanides, actinides, and radioactive elements. Most of these compounds are discovered in experiments, and the remaining ones are predicted by theoretical calculations. The PDOS dataset is attained by the standard DFT calculations with the PBE exchange-correlation function.

In order to fairly compare the performance among the CSGN model with different kinds of clusters and loss functions, we conducted comparative experimental tests on predicting PDOS. We representatively selected seven types of multi-cluster configurations as shown in Figure S1(a) and three kinds of loss functions as depicted in Figure S1(b) from all our test results. The testing for multiple cluster representation parameters primarily considers their ability to cover a wider range of local cluster configurations and equivalent clusters caused by their symmetry. It can be observed that the predictive performance with single type of cluster is significantly inferior, while those with different compositions of three or four cluster types are similar. The best performance is acquired with the four mixing types of clusters with 3, 4, 6, 9 atoms. Moreover, by examining the prediction performance with loss functions constructed from the MAE, MSE, and DTW components, it is found that the best result was achieved with the mixed DTW and MAE loss functions.

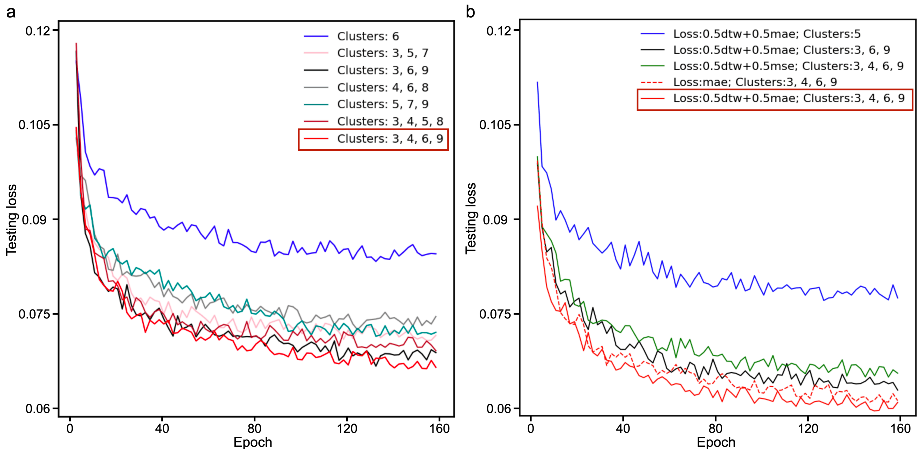


Figure S1. Testing results for (a) different kinds of clusters, and (b) different loss function with the same datasets.

According to the vibrational theory, the CSGN model predicts the total PDOS as a

weighted average of the partial PDOS contributed by multiscale atomic clusters. In the process of constructing crystal graph, one vibrational mode may be double counted by clusters with different sizes or by clusters with different central atoms. If the superposition calculation of all partial DOS is performed directly, it will inevitably lead to incorrect strength distribution of phonon vibration states. For example, the 3-atom, 6-atom and 9-atom clusters in some materials may encompass the same 3-atom vibrational cluster, and therefore the predicted peaks associated with this 3-atom vibrational cluster will be erroneously exaggerated if the contribution of all clusters are directly added up.

To overcome this problem, we implemented two operations in the CSGN model: (1) In the capsule module, we designed a feature $clu_{i,k}^{p}$ in the (0,1) range for each cluster to describe the weight of its contribution to the overall PDOS. The $clu_{i,k}^{p}$ is calculated through a one-layer MLP using the material chemical environment as input and continuously updated during the model training. While the double counting may occur in lots of materials, the effects should be similar for materials with similar structures. Hence, the learning process could drive the feature $clu_{i,k}^{p}$ towards the appropriate values, and eventually the double counting problem is solved through the intelligent reduction of relevant cluster weights. (2) We designed a loss function based on the DTW mechanism to describe the geometric distinctions between spectra. This loss function is sensitive to the variation of peak values, and thus magnifies the loss stemming from the double counting problem compared to traditional MAE. In all, by combining the above operations through iterative training, the contribution weight of each cluster can be accurately learned for the prediction of complex spectra.

## Section 2: Detailed explanation of model architectures

The feedforward propagation frameworks of the multi-scales cluster representation in the cluster encoder (CE) block are summarized in Figure S2. The CE block consists of four modules, including the atom, bond, mass, and capsule-based modules, to build the multi-scales cluster representation.

For the $k$-scale vibrational cluster with the $i^{\text{th}}$ atom serving as central atom, the prior input datasets of the bond module are the initial atom embedding vectors $x_k^i$ (with ($k$×128) dimensions), the atom mass vectors $m_k^i$ (with ($k$×1) dimensions), the bond index vectors $l_k^{index}$ (with ($j$×2) dimensions), and the initial bond vectors $l_k^i$ (with ($j$×128) dimensions), wherein $k$ and $j$ are numbers of atom and bond respectively in each target cluster. The atom embedding and mass vectors describe the type and mass of atoms. The bond index vectors denote the indices of two relevant atoms within each bond. The initial bond vectors represent the bond length of each bond through Gaussian transformation. As illustrated in Figure S2(a), the bond graph vectors (with ($j$×128) dimensions) and the associated cluster mass vectors (with ($j$×1) dimensions) are generated from the atom embedding and atom mass vectors through the bond-level

operations. The bond graph vectors represent the atom arrangement adapted to bond configurations. For the bond connecting the $i^{\text{th}}$ and $j^{\text{th}}$ atoms, the initial bond embedding vector $e_{ij}^{0}$ with 128 dimension is computed as the interatomic distance $|r_{ij}|$ expanded with the Gaussian basis and centered at a series of points $r_n$:

$$e_{ij}^{0} = \exp\left(-\left(|r_{ij}| - r_n\right)^2/\sigma^2\right) \qquad S1$$

wherein the hyper-parameters $\sigma$ is set to 0.5, and $r_n$ is set to a 128-dimensional vector reflecting the equidistant sampling in the range of [0.0, 6.0] with the 6.0 chosen to cover the maximum bond length in this material. Given the initial representation of bond configuration, which is subsequently updated by the MLP layers (with 192 neural nodes) and the Softplus function to provide the updated bond vectors (with ($j$×192) dimensions).

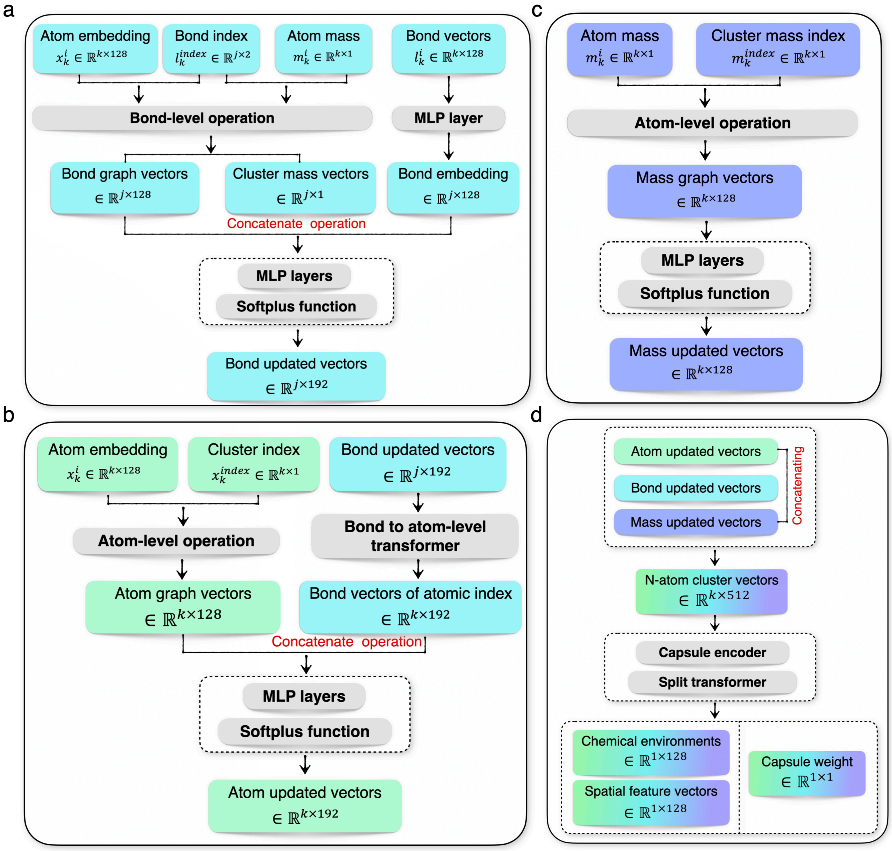


Figure S2. Detailed architecture of the cluster encoder (CE) block comprised of (a) the bond module, (b) the atom module, (c) the mass module, and (d) the capsule encoder module.

The atom module receives the updated bond vectors (with ($j$×192) dimensions), the initial atom embedding vectors (with ($k$×128) dimensions) and the atom index vectors (with ($k$×1) dimensions) to build atom graph representation of each cluster as shown in Figure S2(b). Specifically, the atom graph vectors (with ($k$×128) dimensions) are generated from the initial atom embedding vectors and the atom index vectors through the atom-level operation. The updated bond vectors are transferred to the atom-based

representation via the bond-to-atom transformer. The concatenation of atom graph vectors and bond vectors gives the initial representation of atom configuration, which is subsequently updated by the MLP layers (with 192 neural nodes) and the Softplus function to provide the updated atom vectors (with ($k$×192) dimensions) for each cluster. Similarly, the updated mass vector (with ($k$×128) dimensions) of each cluster is built from the atom mass vector and atom index vectors through atom-level operation in the mass module (Figure S2(c)).

The capsule transformer uses explicitly parameterized affine transformations (mainly including two rotations, two translations, scale and shear) that allow the encoders' inputs to be explained with a small set of transformed objects. It can preserve the general equivariance as it meets the condition of $F\big(T(x)\big) = T'F(x)$, wherein $F$ is a neural network, $x$ is an input object, $T$ and $T'$ are the transformations of the same symmetry group. In the CSGN model, the equivariance relation of the capsule transformer for each cluster $c_{i,k}$ under arbitrary spatial transformations is written as $F_{cap}\big(Mc_{i,k} + t\big) = M'F_{cap}\big(c_{i,k}\big) + t'$, where $F_{cap}$ is the capsule transformer networks in the cluster encoder block. For the spatial transformations $\{t, t', M, M'\} \in E(n)$, n is the space dimension, $t$ and $t'$ are arbitrary translation vectors from the same symmetry group, $M$ and $M'$ are arbitrary orthogonal matrices to represent rotation, mirror, and inversion reflections from the same symmetry group. During the network training, the capsule transformer intelligently finds the spatial transformations that maximize the similarity of chemical environments among different clusters, and updates the chemical environments based on cluster similarity. That means the equivalent clusters stemming from any crystal symmetry would eventually be projected to identical cluster capsule vectors through the capsule transformer. As a result, even without the spherical harmonics or tensor product, our CSGN model based on the capsule networks accurately perceives the equivalence among clusters arising from all symmetry transformations.

The cluster vectors (with ($k$×512) dimensions) is established by the capsule encoder from the updated atom, bond, and mass vectors, and then deconstructed by the split transformer into the cluster capsule representation comprised of the geometric transformations $clu^{s}_{i,k}$ (with (1×128) dimensions), the cluster chemical environment $clu^{c}_{i,k}$ (with (1×128) dimensions), and the presence value $clu^{p}_{i,k}$ (with (1×1) dimensions). The presence value is calculated by a simple neural network layer representing the contribution of the cluster to the overall PDOS, the weight of the cluster with repeated counts will decrease.

The above bond embedding vectors are used as input data for generating the cluster representation $c_{i,k}$ ($i^{\text{th}}$ atom with k-1 neighbor atoms). The CE block perceives the input data of atoms $x^{i}_{k}$, bonds $l^{i}_{k}$ and mass data $m^{i}_{k}$ to construct the initial cluster representation $c_{i,k}$ and cluster capsules $clu^{s}_{i,k}, clu^{c}_{i,k}, clu^{p}_{i,k}$ of target material, eventually takes the form

$$c_{i,k} = f_{cap}(f_{cluster}(x_k^i, l_k^i) \oplus f_{mass}(m_k^i)) \quad (S2)$$

wherein $\oplus$ denotes the concatenation operation, $f_{cluster}$ and $f_{mass}$ are the graph-based networks, $f_{cap}$ is the capsule-based networks. The bond embedding operation within the $f_{cluster}$ network projects the bond configuration vector $l_k^i$ to bond embedding vector $e_{ij}^0$ such that the atom, bond and mass vectors have identical dimension. The entire process of generating cluster representation including the bond embedding operation contains only node-to-node mappings, and does not involve pooling, averaging or dimensionality reduction. This means the cluster representation inherits all the structural equivalence under symmetry operations of the material. Therefore, the equivariant and invariance characteristics of our model arising from the capsule network will be strictly preserved. The corresponding likelihood function takes the form of

$$\mathcal{L}_{CE} = \prod_{m} \prod_{k}^{K} \prod_{i}^{I} P(clu_{i,k}^{s}, clu_{i,k}^{c}, clu_{i,k}^{p} | x_k^i, l_k^i, m_k^i)_m \quad (S3)$$

where $m$ is the index of material, $M$ is the number of crystals in dataset, $i$ is the index of central atom, $I$ is the number of atoms within the primitive cell, $k$ is atom numbers in clusters, K = {3, 4, 6, 9} is the cluster scales selected in this study.

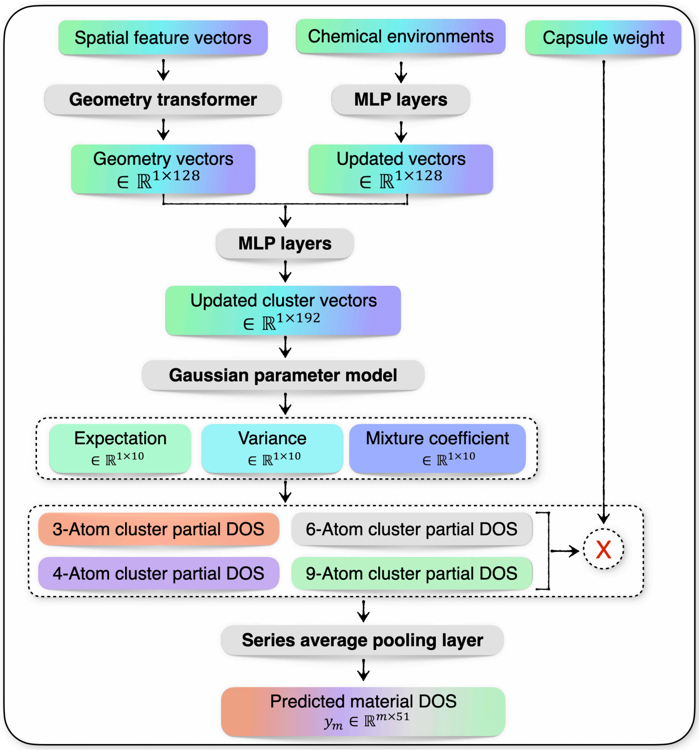

Figure S3. Detailed architecture of the spectrum predictor (SP) block.

The spectrum predictor (SP) block receives the geometric transformations $clu_{i,k}^{s}$ and chemical environments $clu_{i,k}^{c}$ of cluster capsules to construct the updated cluster vectors (with (1×192) dimensions) for each cluster (Figure S3). The key parameters (mean $\mu_{i,k}$, variance $\sigma_{i,k}$, and mixing coefficient $\alpha_{i,k}$) are calculated by the Gaussian

parameter model $\Theta_g()$, which consists of MLP layers with different activation functions (Softmax) to extract statistic features from the updated cluster vectors. The partial PDOS (with 256 dimensions) for each cluster capsule is attained by uniform sampling from the relevant mixed Gaussian distribution. The predictive PDOS $y_m$ (with (m×51) dimensions) is eventually obtained by linear superposition of all partial PDOS through series average pooling with capsule weight $clu_{i,k}^{p}$,

$$y_{1\to t} = \sum_{k}\sum_{i=1}^{I} clu_{i,k}^{p} \cdot (\sum_{j=1}^{n} \mu_j(c_{i,k}) \cdot \mathcal{N}\left(\theta_j(c_{i,k}), \sigma_j^2(c_{i,k})\right)) \qquad (S4)$$

wherein $\theta_j()$, $\sigma_j()$, and $\mu_j()$ are the fitting function of mean value, covariance, and weight parameter of $j^{\text{th}}$ Gaussian process, which are calculated by the corresponding MLP layers. After appropriate model training, the unnecessary peaks will be eliminated as the related weight parameter $\mu_j(c_{i,k})$ gradually drops to zero.

In the CSGN model, the MGP process for the cluster $c_{i,k}$ should generate the partial PDOS spectrum, such that the number of Gaussian peaks is equal to the number of vibrational modes related to the cluster $c_{i,k}$. Therefore, it is very important to ensure that the hyperparameter n exceed the maximum number of vibrational modes that can be generated by each cluster. We safely selected the value n=10, because most of the materials in our dataset possess less than a total of 10 peaks arising from multiple vibrational clusters. Setting n as a too small value would reduce the model performance owning to the insufficient representation of vibrational modes, while picking a too large value would induce additional overfitting problem.

The likelihood function of the SG block can be written as

$$\mathcal{L}_{SG} = \prod_{m}^{M}\sum_{k}^{K}\sum_{i}^{I} P(y_{1\to t}^{real} | clu_{i,k}^{p} \times \Theta_g(\emptyset_{cap}(clu_{i,k}^{s}, clu_{i,k}^{c}))) \qquad (S5)$$

wherein $y_{1\to t}^{real}$ is the ground truth value of PDOS for the $m^{\text{th}}$ material, $\emptyset_{cap}$ is a set of capsule-based algorithms in the SP block.

The likelihood function describing the whole feedforward propagation process from material input data to property prediction can be obtained through the production of likelihood functions related to the two subprocess.

$$\mathcal{L} = \prod_{m}^{M}\prod_{k}^{K}\prod_{i}^{I}[P(y_m | \emptyset_{SP}(c_{i,k}))P(c_{i,k} | x_k^i, l_k^i, m_k^i)] \qquad (S6)$$

wherein $c_{i,k} = \{clu_{i,k}^{s}, clu_{i,k}^{c}, clu_{i,k}^{p}\}$, and $\emptyset_{SP}()$ is the short for all models of SP process. The loss function ($\mathcal{L}$) was set as the summation of DTW discrepancy and mean absolute errors (MAE):

$$\mathcal{L} = min\langle A, \Delta(y_{1\to t}, y_{1\to t}^{real})\rangle + \frac{1}{M}(\sum_{m=1}^{M} |y_{1\to t} - y_{1\to t}^{real}|) \qquad (S7)$$

wherein $A$ is the alignment matrix to compute the optimal alignment path for two series, $\Delta(\cdot)$ is the cost matrix by calculating cumulative distance between predicted PDOS $y_{1\to t}$ and real PDOS $y_{1\to t}^{real}$.

**Section 3: Contributions of vibrational clusters on the PDOS spectrum of $BaPdF_4$ crystal.**

In order to examine the contribution of each vibration mode to PDOS spectrum, the 2-sample Kolmogorov-Smirnov (2-KS) was calculated between the partial spectra and the four spectral peaks of the $BaPdF_4$ crystal predicted by the CSGN model (Figure 2b in the main test). The normalized 2-KS values reflecting the contribution of each cluster to certain PDOS peaks were documented in Table S1. There is only one inequivalent cluster given specific cluster scale and central atom in $BaPdF_4$ due to its high crystal symmetry.

Table S1. Normalized 2-KS between the partial spectra and the four spectral peaks predicted by CSGN, along with the cluster information.

| Cluster scale k | Central atom | Normalized 2-KS of predicted phonon spectrum | | | |
|---|---|---|---|---|---|
| | | Peak 1 | Peak 2 | Peak 3 | Peak 4 |
| k=3 | Ba | 0.92 | 0.08 | 0 | 0 |
| | Pd | 0 | 0 | 0 | 1 |
| | F | 0.23 | 0 | 0.42 | 0.35 |
| k=4 | Ba | 0.96 | 0.04 | 0 | 0 |
| | Pd | 0.10 | 0 | 0 | 0.90 |
| | F | 0.35 | 0.65 | 0 | 0 |
| k=6 | Ba | 0.37 | 0.63 | 0 | 0 |
| | Pd | 0.34 | 0.66 | 0 | 0 |
| | F | 0.64 | 0.36 | 0 | 0 |
| k=9 | Ba | 1 | 0 | 0 | 0 |
| | Pd | 1 | 0 | 0 | 0 |
| | F | 0.58 | 0.42 | 0 | 0 |

## Section 4: Global trends of the predicted PDOS spectra on cluster mass and bond length.

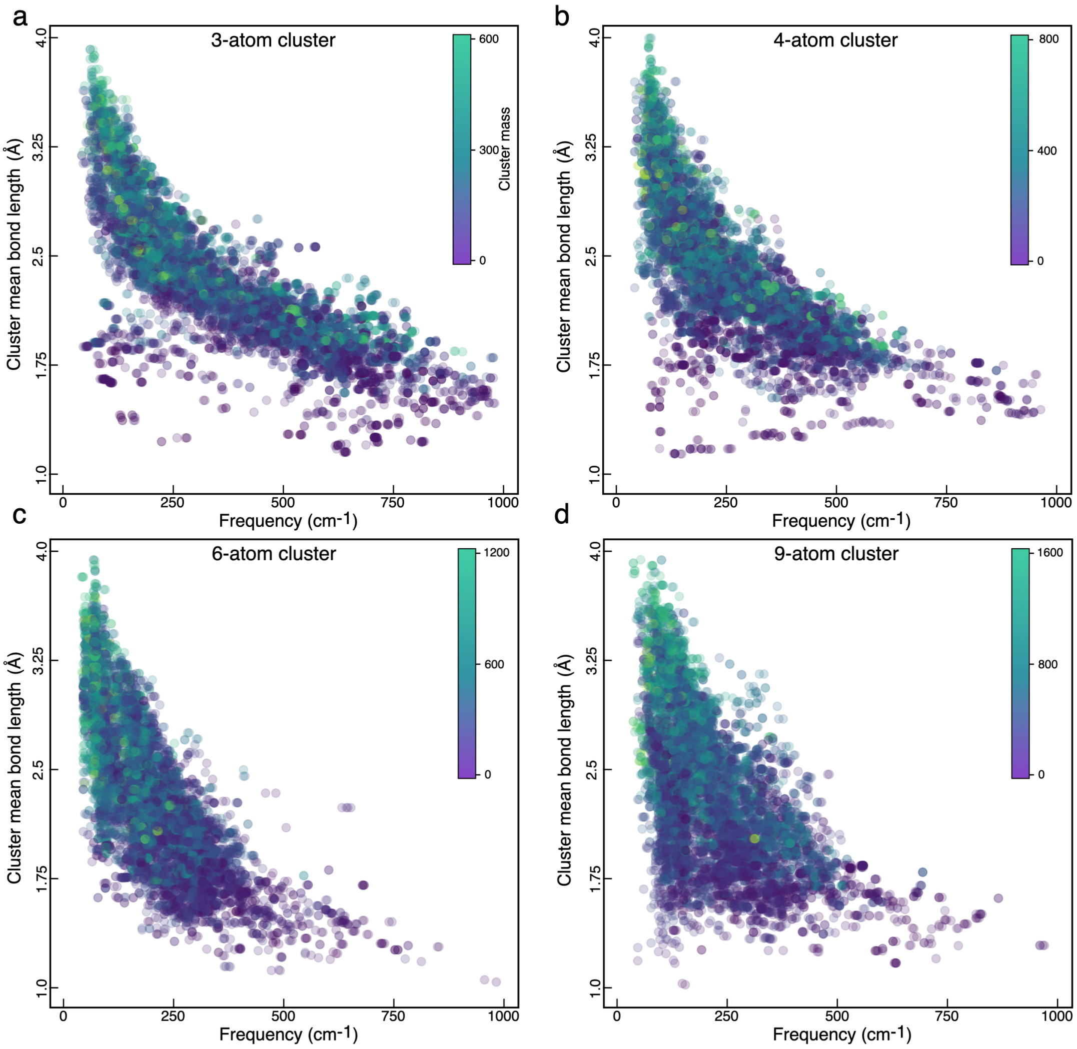


Figure S4. Correlation analysis among cluster mean bond length, cluster mass, and major peak frequency for (a) 3-atom clusters, (b) 4-atom clusters, (c) 6-atom clusters, and (d) 9-atom clusters, with the cluster mass illustrated by the color of nodes.

For individual clusters in all materials, the frequencies of the main peak in the predicted partial PDOS spectra were extracted and collectively presented in Figure S4, with each panel incorporating the vibration clusters with uniform scale (k = 3, 4, 6, and 9). The results imply that our cluster representation is capable of generating diverse PDOS spectra to continuously cover the entire energy range of relevant phonon modes. The overall trend that the peak frequency increases with decreasing cluster mass and average bond length can be found in the peak distributions, which is consistent with the vibrational origin of phonon spectrum.

To quantify the strain effect in our model, we have conducted two series of additional calculations for predicting PDOS with material datasets in different strain

conditions, with the focuses on demonstrating the robustness and physical interpretability of the CSGN model.

(1) We applied random strain in one direction or three directions to all materials in the dataset, with a strain range of (-1%, 1%) and (-2%, 2%). After retraining the CSGN model, the MAE errors for predicting the PDOS in the three tests (with random 1% uni-directional (Figure S5(a)), 2% uni-directional (Figure S5(b)), and 1% triple-directional strains (Figure S5(c))) became 0.066, 0.068, and 0.071, which slightly increased compared to that of the original model (0.061) but still outperformed other works. Further, the predicted performance of the total number of phonon states were examined under the three tests as shown in Figure S5, which exhibited similar distributions to the original model. These results verified the robustness of CSGN model to strain effect.

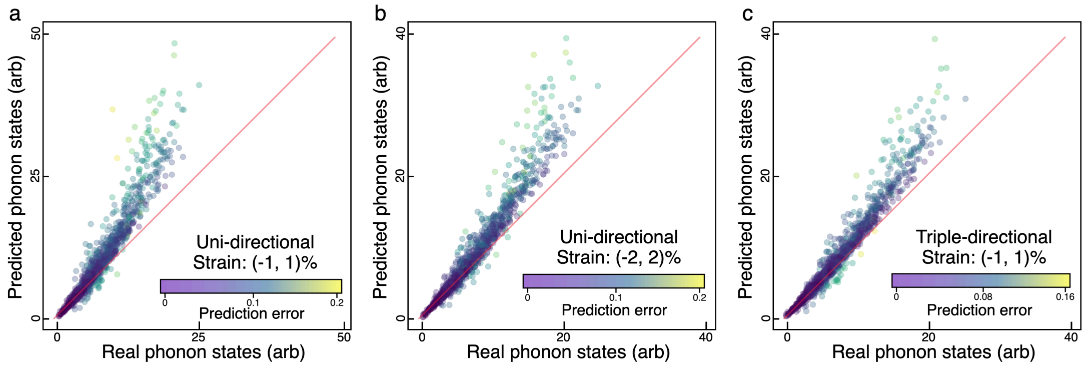


Figure S5. Prediction performances of phonon state numbers by the CSGN with (a) random uni-directional strain (-1%, 1%), (b) random uni-directional strain (-2%, 2%) and (c) random triple-directional strain (-1%, 1%).

(2) In order to confirm that the intrinsic correlation between bond length (strain) and PDOS are correctly learned by our model, we predicted the PDOS of representative materials under different strains, as illustrated in Figure S6. For all tested materials, the main peak positions of the PDOS gradually moved towards low frequency region with the strain changing from -4% to +4%. This can be explained by the peak frequency slightly drops with increasing bond lengths, and indicated that the CSGN model successfully learned the underlying structure-property relation.

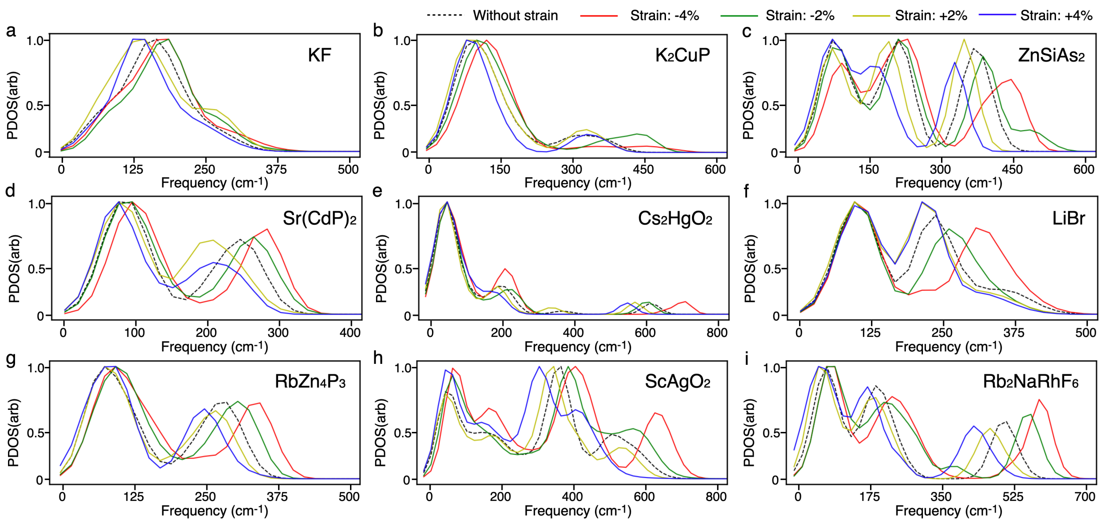


Figure S6. Comparison of predictive PDOS spectra with different strain for nine representative materials.

## Section 5: Verification of cluster correlation learned by CSGN model.

The correlations among clusters with the same components but in different crystal environments were examined by calculating the Pearson coefficients between partial PDOS for the O-Ti-O and O-Zn-O cluster groups in 36 representative materials, as shown in the chord diagram in Figure 3c of the main text. Examples of the calculated Pearson coefficients are presented in Table S2, and the complete information regarding the chord diagram is documented in the source data file.

Table S2. The Pearson correlations between cluster partial PDOS.

| Material | O-Ti-O | | | | |
|---|---|---|---|---|---|
| | $TiCdO_3$ | $TiCdO_3$ | BaTiO3 | $BaTiO_3$ | $BaTiO_3$ |
| $TiCdO_3$ | 1 | 0.996 | 0.823 | 0.800 | 0.982 |
| $TiCdO_3$ | 0.996 | 1 | 0.844 | 0.822 | 0.987 |
| $BaTiO_3$ | 0.823 | 0.844 | 1 | 0.991 | 0.862 |
| $BaTiO_3$ | 0.801 | 0.822 | 0.991 | 1 | 0.854 |
| $BaTiO_3$ | 0.982 | 0.987 | 0.862 | 0.854 | 1 |

The correlations between clusters with different atomic components depend on a set of complicated features, including the cluster components, structure, mass, bond distribution, electronegativity, and local chemical environment. The information of vibrational clusters relevant to the discussion in the main text is documented in Table S3-S6. Correlation analysis among cluster mean bond length, cluster mass, and major

peak frequency for (a) $XO_2$, (b) $XO_3$, (c) $XO_5$, and (d) $YO_8$ clusters (X = Cd, Ag, Zn, Ti, and Al; Y = Cs, Ba, Sr, Cd, and K elements) is presented Figure S7.

Table S3. The prior information of Ba-X-Y-O clusters in different materials.

| Cluster | Material | X element | Bond length (Å) | Mass (Da) | Electronegativity | Peak position ($cm^{-1}$) |
|---|---|---|---|---|---|---|
| $XO_2$ | $Ba_2YSbO_6$ | Y | 2.21 | 88.9 | 1.22 | 400 |
| | $Ba_2LaNbO_6$ | Nb | 2.01 | 92.9 | 1.60 | 550 |
| | $Ba_2YsbO_6$ | Sb | 1.99 | 121.8 | 2.05 | 650 |
| | $Ba_2LiReO_6$ | Re | 1.87 | 186.2 | 1.90 | 900 |
| $XO_3$ | $Ba_2LaSbO_6$ | La | 2.34 | 138.9 | 1.10 | 420 |
| | $Ba_2LaNbO_6$ | La | 2.37 | 138.9 | 1.10 | 400 |
| | $Ba_2LaTaO_6$ | La | 2.37 | 138.9 | 1.10 | 360 |
| | $Ba_2YsbO_6$ | Sb | 1.99 | 121.8 | 2.05 | 655 |
| $XO_5$ | $Ba_2LiReO_6$ | Re | 1.88 | 186.2 | 1.90 | 180 |
| | $Ba_2LaNbO_6$ | La | 2.36 | 138.9 | 1.10 | 260 |
| | $Ba_2LaSbO_6$ | Sb | 2.01 | 121.8 | 2.05 | 280 |
| | $Ba_2YsbO_6$ | Y | 2.21 | 88.9 | 1.22 | 380 |
| $BaO_8$ | $Ba_2LiReO_6$ | Ba | 2.86 | 137.3 | 0.89 | 120 |
| | $Ba_2LaNbO_6$ | | 2.85 | | 0.89 | 120 |
| | $Ba_2LaSbO_6$ | | 2.89 | | 0.89 | 120 |
| | $Ba_2LaTaO_6$ | | 2.84 | | 0.89 | 120 |

Table S4.The symmetry information of crystal materials for Ba-X-Y-O group.

| Material | Crystal system | Space group |
|---|---|---|
| $Ba_2YsbO_6$ | Cubic | *Fm-3m* |
| $Ba_2LaTaO_6$ | Monoclinic | *C2/m* |
| $Ba_2YsbO_6$ | Trigonal | *R-3* |
| $Ba_2LaNbO_6$ | Monoclinic | *C2/m* |
| $Ba_2LiReO_6$ | Cubic | *Fm-3m* |

Table S5. The prior information of Cs-X-Y-F clusters in different materials.

| Cluster | Material | X element | Bond length (Å) | Mass (Da) | Electronegativity | Peak position ($cm^{-1}$) |
|---|---|---|---|---|---|---|

| | | | | | | |
|---|---|---|---|---|---|---|
| $XF_2$ | $Cs_2LiNF_6$ | N | 1.85 | 14.0 | 3.04 | 720 |
| | $Cs_2KGaF_6$ | Ga | 2.56 | 69.7 | 1.81 | 475 |
| | $Cs_2KInF_6$ | In | 2.09 | 114.8 | 1.78 | 400 |
| | $Cs_2KTlF_6$ | Tl | 2.17 | 204.4 | 2.04 | 380 |
| $XKF_2$ | $Cs_2KGaF_6$ | Ga | 2.39 | 69.7 | 1.81 | 280 |
| | $Cs_2KRhF_6$ | Rh | 2.43 | 102.9 | 2.28 | 270 |
| | $Cs_2KInF_6$ | In | 2.52 | 114.8 | 1.78 | 200 |
| | $Cs_2KTlF_6$ | Tl | 2.58 | 204.4 | 2.04 | 180 |
| $XF_5$ | $Cs_2LiNF_6$ | N | 1.85 | 14.0 | 3.04 | 480 |
| | $Cs_2KGaF_6$ | Ga | 1.91 | 69.7 | 1.81 | 410 |
| | $Cs_2KInF_6$ | In | 2.09 | 114.8 | 1.78 | 275 |
| | $Cs_2KTlF_6$ | Tl | 2.17 | 204.4 | 2.04 | 185 |
| $CsF_8$ | $Cs_2KGaF_6$ | Ga | 3.17 | 69.7 | 1.81 | 85 |
| | $Cs_2KRhF_6$ | Rh | 3.19 | 102.9 | 2.28 | 85 |
| | $Cs_2KInF_6$ | In | 3.27 | 114.8 | 1.78 | 60 |
| | $Cs_2KTlF_6$ | Tl | 3.32 | 204.4 | 2.04 | 60 |

Table S6. The symmetry information of crystal materials for Cs-X-Y-F group.

| Material | Crystal system | Space group |
|---|---|---|
| $Cs_2LiNF_6$ | Cubic | *Fm-3m* |
| $Cs_2KGaF_6$ | Cubic | *Fm-3m* |
| $Cs_2KInF_6$ | Cubic | *Fm-3m* |
| $Cs_2KTlF_6$ | Cubic | *Fm-3m* |
| $Cs_2KRhF_6$ | Cubic | *Fm-3m* |

Table S7. The bond information for the Ba-X-Y-O and Cs-X-Y-F cluster groups.

| Ba-X-Y-O group | | | Cs-X-Y-F group | | |
|---|---|---|---|---|---|
| Bond | Bond energy (kJ/mol) | Bond type | Bond | Bond energy (kJ/mol) | Bond type |
| Y-O | 300-400 | Ionic bond | N-F | 150-250 | Covalent bond |
| Nb-O | 600-800 | Ionic bond | Ga-F | 300-400 | Covalent bond |
| Sb-O | 200-300 | Covalent bond | In-F | 200-300 | Covalent bond |
| Re-O | 300-400 | Covalent bond | Tl-F | 400-500 | Ionic bond |

| | | | | | |
|---|---|---|---|---|---|
| La-O | 200-300 | Ionic bond | Rh-F | 400-500 | Covalent bond |

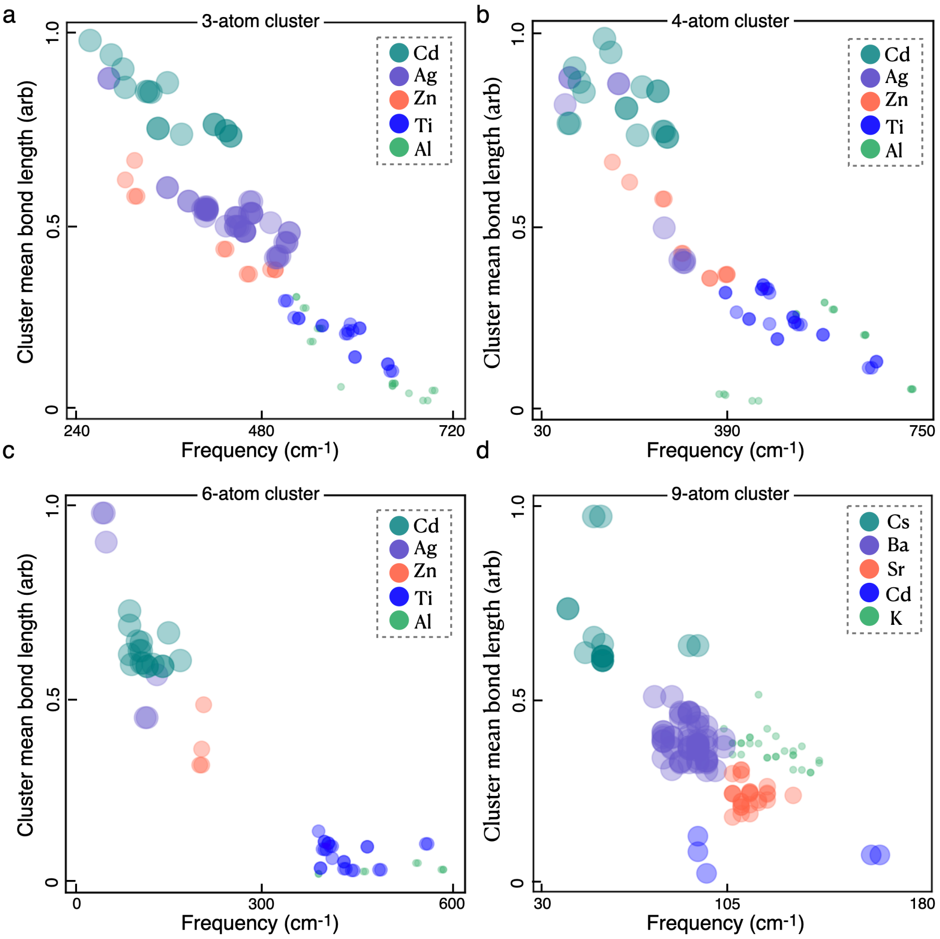


Figure S7. Correlation analysis among cluster mean bond length, cluster mass, and major peak frequency for (a) $XO_2$, (b) $XO_3$, (c) $XO_5$, and (d) $YO_8$ clusters (X = Cd, Ag, Zn, Ti, and Al; Y = Cs, Ba, Sr, Cd, and K elements). Atom type and cluster mass are illustrated by the color and size of nodes respectively.

## Section 6: Prediction performance of the CSGN model

The predicted results of PDOS spectra for all materials in the above cluster correlation analyses ($Ba_2LiReO_6$, $Ba_2LaNbO_6$, $Ba_2LaSbO_6$, $Ba_2YSbO_6$, $Ba_2LaTaO_6$, $Cs_2LiNF_6$, $Cs_2KGaF_6$, $Cs_2KRhF_6$, $Cs_2KTlF_6$, and $Cs_2KInF_6$) are presented in the Figure S8. The ground truth data and the predicted spectra by CSGN with DTW are shown in the black dashed line and blue line, respectively.

In addition, the performance comparison among different models (CSGN without and with DTW kernel, and Mat2Spec model[2]) were investigated for 12 materials as shown in Figure S9. In order to validate the transferability of CSGN model, complicated ternary and quaternary materials, with no similar samples composed of identical

elements in the training set, were examined as shown in Figure S9(g)-S9(l). The results suggest that our CSGN model possesses the best prediction accuracy and spectral generation capability.

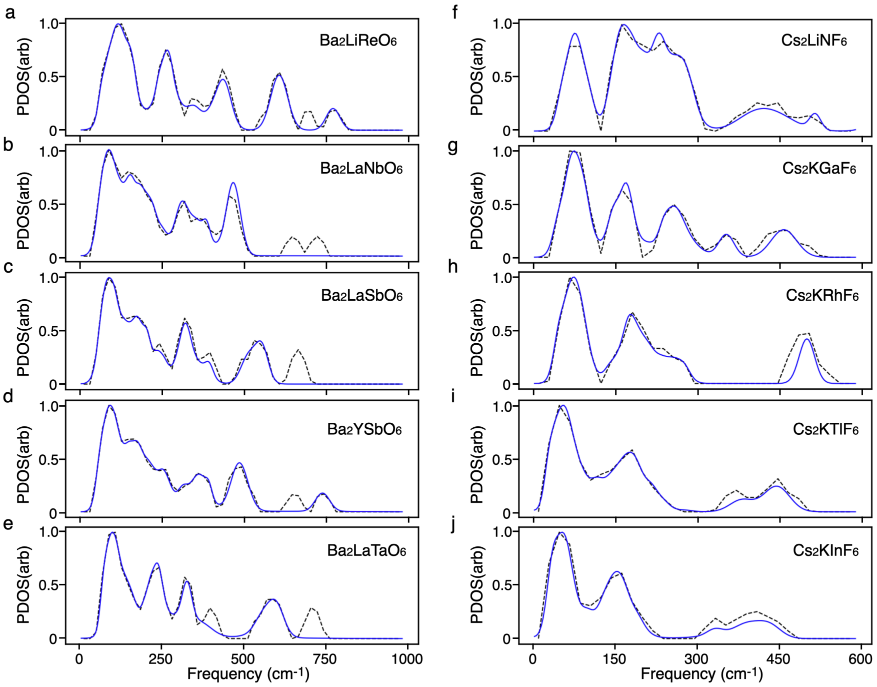


Figure S8. Prediction performance for the Ba-X-Y-O groups and the Cs-X-Y-F groups. The ground truth data and the predicted spectra by CSGN with DTW are shown in the black dashed line and blue line, respectively.

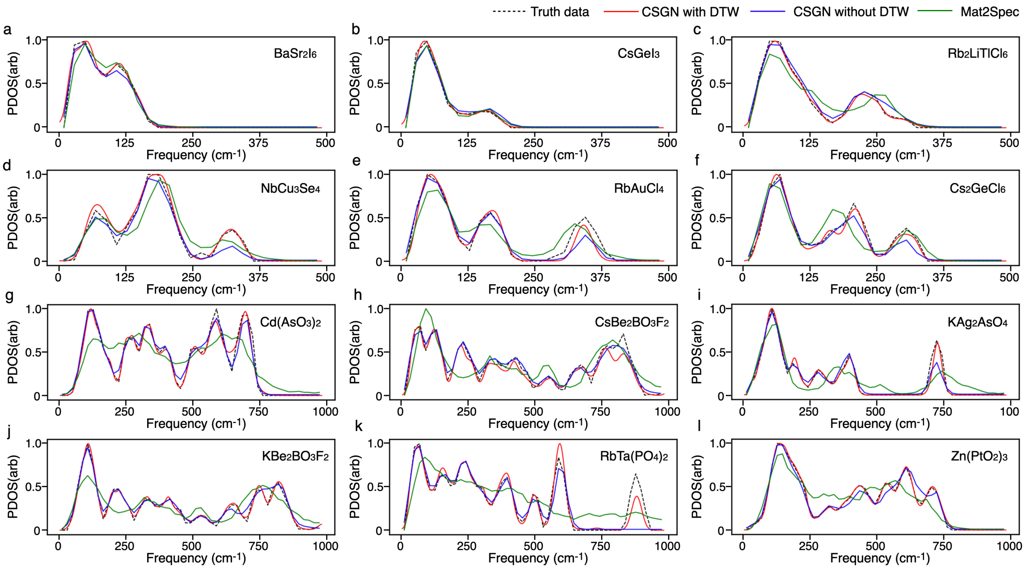


Figure S9. Comparison of prediction performance among different models for 12 materials (a-l). The ground truth data and predicted data by CSGN without and with DTW (dynamic time warping) kernel, and Mat2Spec model are presented in black dashed line, blue line, red line, and green line respectively.

## Supporting Reference

[1] C. Liang, Y. Rouzhahong, C. Ye, C. Li, B. Wang, H. Li *Nat. Commun*. **2023**, *14*, 5198.

[2] S. Kong, F. Ricci, D. Guevarra, J. B. Neaton, C. P. Gomes, J. M. Gregoire. *Nat. Commun.* **2022**, *13*, 949.
